# An Exploratory Analysis of the Neural Correlates of Human-Robot Interactions With Functional Near Infrared Spectroscopy

**DOI:** 10.3389/fnhum.2022.883905

**Published:** 2022-07-18

**Authors:** Emre Yorgancigil, Funda Yildirim, Burcu A. Urgen, Sinem Burcu Erdogan

**Affiliations:** ^1^Department of Medical Engineering, Acibadem Mehmet Ali Aydinlar University, Istanbul, Turkey; ^2^Cognitive Science Master's Program, Yeditepe University, Istanbul, Turkey; ^3^Department of Computer Engineering, Yeditepe University, Istanbul, Turkey; ^4^Department of Psychology, Bilkent University, Ankara, Turkey; ^5^Neuroscience Graduate Program, Bilkent University, Ankara, Turkey; ^6^Aysel Sabuncu Brain Research Center, National Magnetic Resonance Research Center (UMRAM), Ankara, Turkey

**Keywords:** human-robot interaction, face-voice matching, prefrontal cortex, functional near infrared spectroscopy (fNIRS), hemodynamic

## Abstract

Functional near infrared spectroscopy (fNIRS) has been gaining increasing interest as a practical mobile functional brain imaging technology for understanding the neural correlates of social cognition and emotional processing in the human prefrontal cortex (PFC). Considering the cognitive complexity of human-robot interactions, the aim of this study was to explore the neural correlates of emotional processing of congruent and incongruent pairs of human and robot audio-visual stimuli in the human PFC with fNIRS methodology. Hemodynamic responses from the PFC region of 29 subjects were recorded with fNIRS during an experimental paradigm which consisted of auditory and visual presentation of human and robot stimuli. Distinct neural responses to human and robot stimuli were detected at the dorsolateral prefrontal cortex (DLPFC) and orbitofrontal cortex (OFC) regions. Presentation of robot voice elicited significantly less hemodynamic response than presentation of human voice in a left OFC channel. Meanwhile, processing of human faces elicited significantly higher hemodynamic activity when compared to processing of robot faces in two left DLPFC channels and a left OFC channel. Significant correlation between the hemodynamic and behavioral responses for the face-voice mismatch effect was found in the left OFC. Our results highlight the potential of fNIRS for unraveling the neural processing of human and robot audio-visual stimuli, which might enable optimization of social robot designs and contribute to elucidation of the neural processing of human and robot stimuli in the PFC in naturalistic conditions.

## Introduction

With the rapid advances in robotic technology, automated systems such as social robots and virtual agents have taken an increasing number of roles to facilitate our daily lives. One critical question to be addressed at this stage is, whether neural processing of human-human and human-robot interactions shares the same cerebral physiological mechanisms and if so, how this information could be used to calibrate human reactions during human-robot interactions. This information is valuable as it can help us to improve robotic designs for a more efficient and ergonomic experience during human-robot interactions which, from a neuroscientific perspective, involve complex cognitive mechanisms in terms of social cognition and emotional regulation.

Understanding the neurophysiological mechanisms underlying the human-robot interaction with social robots requires interpretation and quantification of the neural correlates of two important neuropsychological components which are processing of (i) face and (ii) voice stimuli from robots and humans. Robots can trigger emotional responses such as distress, happiness and trust in humans (Breazeal et al., 2016). Appraisal variables of emotion during human-robot interactions involve complex external cues such as face and voice stimuli of the robot as well as human behavioral responses such as internal judgements based on prejudice and former memories. In accordance with the appraisal theory of emotions which state that there is a causal link between cognition and emotion, prefrontal cortex (PFC) has been associated with emotion appraisal and attribution of perceptual clues about face and voice to a decent extent in neuroscience literature (Lazarus, 1991; Forbes and Grafman, 2010).

Within this context, functional near infrared spectroscopy (fNIRS) has gained increasing interest as a mobile imaging technology for interpreting the neural correlates of emotional processing in the human PFC. fNIRS is a relatively novel functional brain imaging modality which enables continuous and real time measurement of the local changes in oxygenated hemoglobin (HbO) and deoxygenated hemoglobin (HbR) concentration levels non-invasively in naturalistic settings. Task induced alterations in neural activity in localized brain regions leads to an increased local metabolic rate of oxygen consumption which results in an increase in local oxygenated blood flow. By use of ergonomic and wearable probes which are placed at the surface of the scalp, fNIRS systems have proven their feasibility in quantification of cerebral hemodynamic activation during various types of cognitive stimuli in numerous studies (Erdogan et al., 2014; Yu et al., 2017). fNIRS has become an increasingly popular and preferred neurophysiological measurement method for a broad band of research areas which ideally require practical access to PFC functional activity such as cognitive psychology, adult and children psychiatry and sports physiology. Some advantages of fNIRS for cognitive neuroscience studies include its robustness to motion artifacts when compared to fMRI and EEG, limited exogenous noise, quick set-up time and calibration, suitability to collect functional information in naturalistic environments and ability to collect data from a broad range of subject populations such as children and elderly adults (Tuscan et al., 2013). Moreover, mobile fNIRS devices can allow monitoring of brain physiology during physical interaction with robots, where the hemodynamic correlates of neural activation can be recorded during behavioral human-robot interaction tasks (Henschel et al., 2020).

Taking into consideration the cognitive complexity of human-robot interactions, the aim of this study was to explore the differences and similarities in perceptual and emotional processing of human and robot faces during a human-robot interaction task. For this purpose, an experimental paradigm which consisted of auditory and visual presentation of human and robot stimuli was designed and hemodynamic activity of the PFC was continuously monitored with an fNIRS system to interpret the spatiotemporal patterns of hemodynamic activity in response to presented stimuli. More specifically, our experimental paradigm involved four different types of audio-visual video stimuli involving different combinations of human and robot face and voice. Hence, it enabled us to interpret the differences in hemodynamic responses obtained during face and voice mismatch conditions which have never been investigated before in a naturalistic setting with fNIRS methodology.

The novelty of our study is employment of the fNIRS method to explore the differences and similarities in hemodynamic activation in response to both face and voice components during a human-robot interaction task and evaluate the hemodynamic correlates of face-voice mismatch situations. Evaluating the correlations between behavioral rating scores and hemodynamic responses to robot and human stimuli may have some profound importance as it may lead us to develop a deeper understanding of the neuro-cognitive mechanisms underlying human-robot interactions. Advancing knowledge on the neurophysiological mechanisms underlying the cognitive and behavioral responses to presentation of robot stimuli may allow us to design more ergonomic and comforting social robots in the future.

## Materials and Methods

### Participants

Thirty-five healthy adults participated in our study. Six participants were excluded from the analysis due to poor quality of the recordings. Remaining 29 participants had an age range between 20 and 38 (M = 28.5, SD = 5.5), which consisted of 11 females and 18 males. All participants had a graduate degree or were undergraduate students. Hence, all participants had digital literacy and were active users of computers in their daily lives.

None of the participants had any neurological or psychiatric disorder during the time of the recordings and they had normal or corrected to normal vision. Participants were informed about the experimental procedure and the fNIRS technique prior to the onset of the experiments. They had read and signed informed consent forms before the onset of the experiment. The study was approved by the Non-Interventional Research Ethics Board of Medipol University, Istanbul, Turkey.

### Audio-Visual Video Stimuli

Audio-visual videos of a real human and a primitive robot design were used as stimuli. Reallusion Character Creator 3® digital design software was used for creating 3D models of primitive robot face videos. Default robot models from this software were reshaped and the inner mechanical mesh of the robot was modified to have a glossy texture skin. Reallusion iClone-7 software was used for speech animation of robot face videos. Text to speech plug-in was used as the basis of speech animation and mouth motion. At the final step, design errors and bugs were checked and fixed manually.

**Figure 1:**
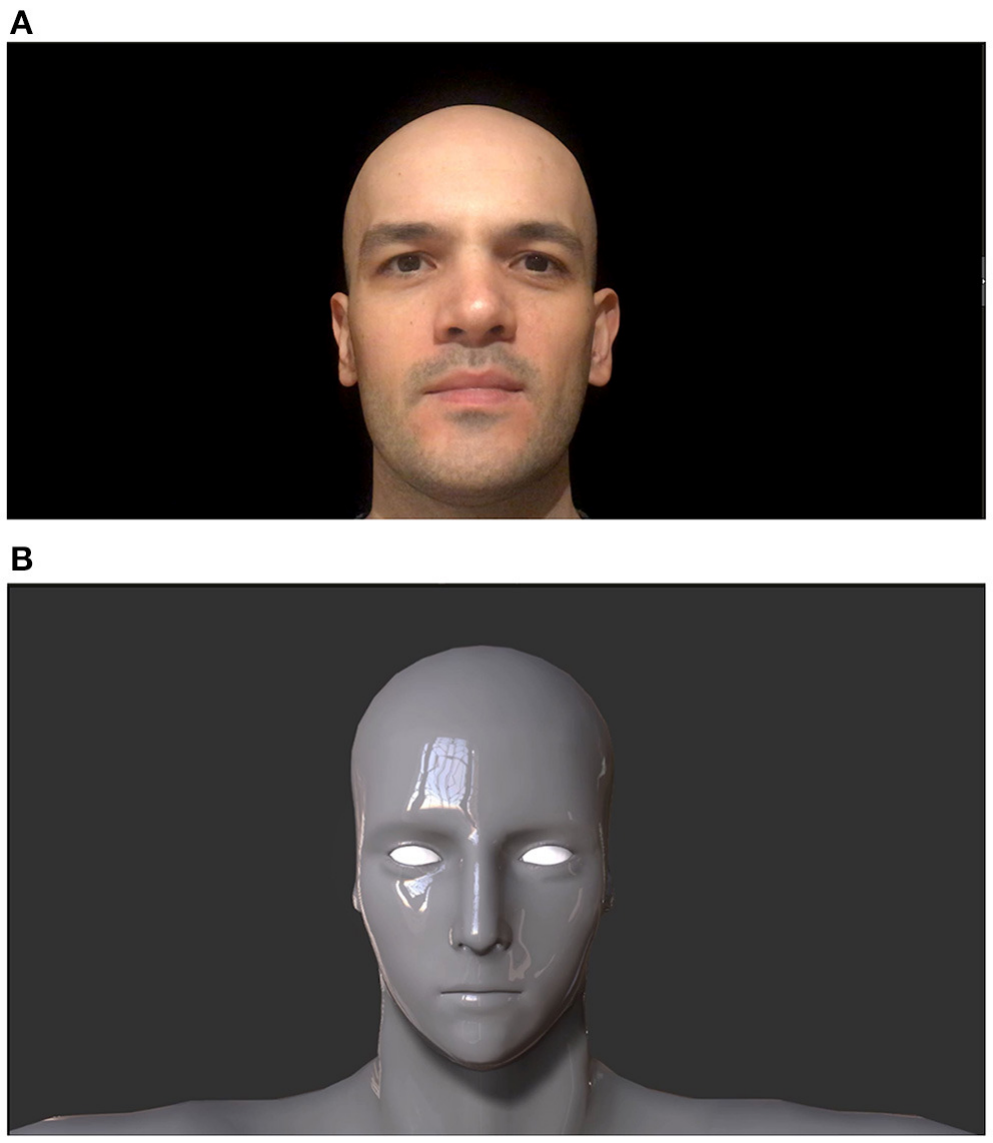
Human **(A)** and robot faces **(B)**. Adapted from Mutlu et al. (2020).

Human face video belonged to a 34-year-old male volunteer. His complete baldness was a common feature with robot face designs and he is white skinned (Figure 1A). The primitive robot design of robot face stimulus did not have facial feature details. Oval shaped basic eyes, mouth line, and a primitive nose were the only components of the robot face. Metallic and glossy skin of the robot was a coherent aspect to be expected from robot designs (Tinwell et al., 2015), (Figure 1B).

Robot voice and human voice were the two types of auditory stimuli presented from a computer monitor in the experiment. The natural voice of a 33-year-old male model, the same model as in the human face stimulus, was used. The human and robot voice stimuli consisted of the phrase “How are you, how is it going?”. The robot voice was derived by processing real human voice as well. This raw sound file was processed in a way where pitch and formant parameters were altered (2 minor 5, 9.48 formant) and a novel high-toned artificial sound (named as robot voice condition) was produced (Yorgancigil et al., 2021).

Experimental stimuli consisted of human and robot faces repeating the above sentence where the faces were matched with either robot voice or human voice. Hence, the 2 types of visual stimuli and 2 types of auditory stimuli were paired together to generate 4 types of audio-visual video stimuli (2 visual: “human face,” “robot face” x 2 audio: “human voice,” “robot voice”) forming either congruent (i.e., human face-human voice, robot face-robot voice) or incongruent face-voice (i.e., human face-robot voice, robot face-human voice) pairs. Auditory and visual stimuli pairs were combined using Adobe Premiere® software as audio-visual videos. Each audiovisual video has a length of 4 s.

### Experimental Design

Audio-visual robot and human face videos were presented to participants in two sessions. In the first session, they only watched the videos on the screen while their PFC hemodynamic activity was continuously recorded with an fNIRS system. In the second session, they watched the stimuli videos again for behavioral assessment and rating the stimuli (described below) immediately after they had finished the first session. This arrangement minimized the effect of fatigue and adaptation during fNIRS recordings in the first session.

The experiment was programmed with the open source PsychoPy3 software. PsychoPy3 is a Python library which has a special builder interface to run visual and auditory cognitive science experiments (Peirce, 2009). Participants sat on a comfortable chair in front of a computer monitor which had a 60 Hz refresh rate, 3,200 x 1,800 resolution and 13 inches size. After instructions were given by the researcher, participants were alone in a silent dark room (Hwang and Lee, 2021). They were asked to keep their eyes open and avoid head movements during the fNIRS recording episode. Potential feelings of discomfort due to the placement of the fNIRS optodes were checked before the onset of the experiment and it was assured that all participants had a comfortable experience.

**Figure 2:**
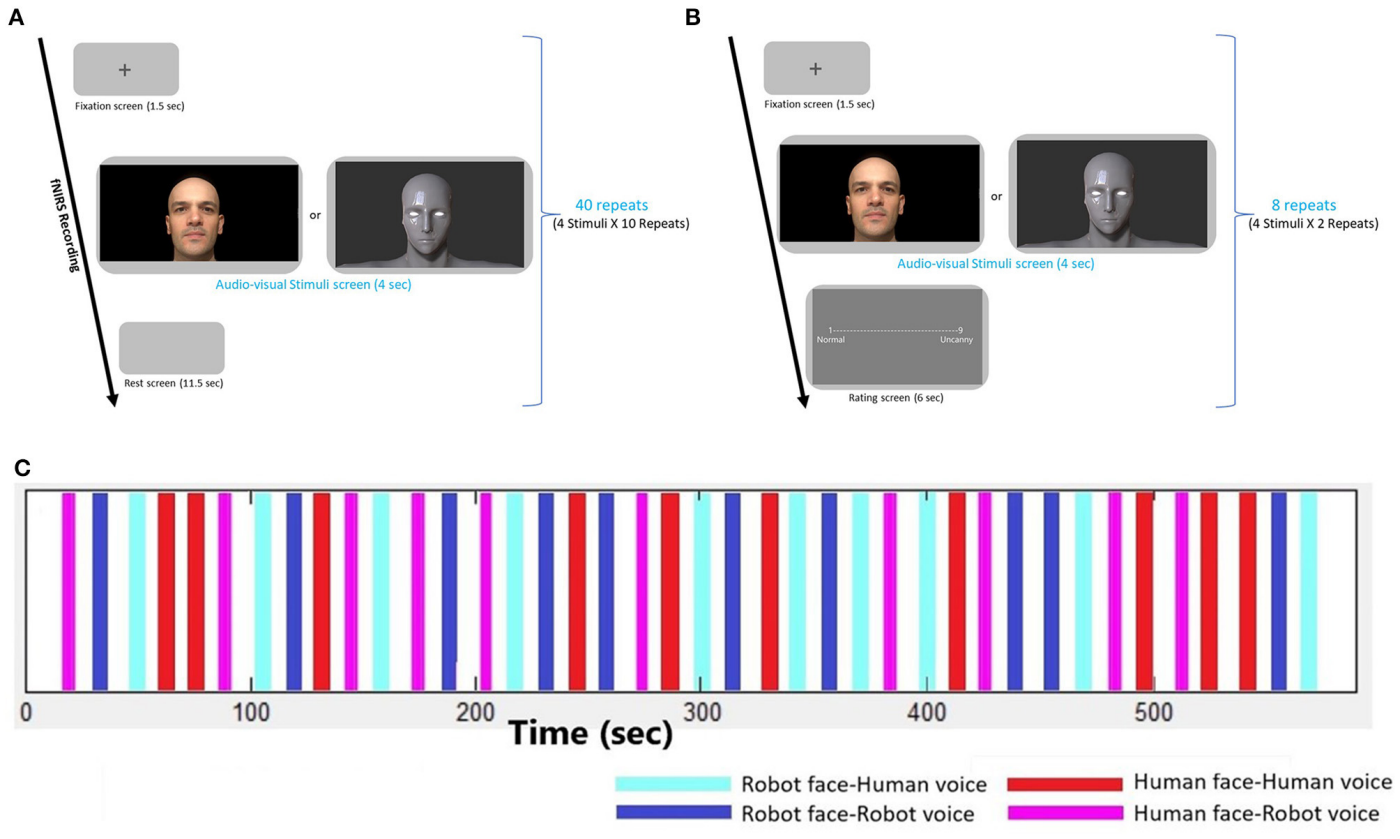
Experimental design. **(A)** Experimental design of the fNIRS recording session, **(B)** Experimental design of the Behavioral session, **(C)** Time traces depicting the onsets and durations of each audio-visual stimulus during the fNIRS recording session. Four types of stimuli are repeated 10 times in a randomized order.

In the first fNIRS recording session, 4 types of robot and human stimuli were presented, each of which lasted for 4 s, followed by a rest period of 13 s. A gray blank screen was presented during the rest periods. The order of stimuli was randomized and each type of stimuli was presented 10 times. Hence, the fNIRS recordings took approximately 12 min for each participant (Figures 2A,C).

The second session of the experiment consisted of a behavioral paradigm. Participants responded to behavioral assessment to evaluate emotional context of audio-visual stimuli. Self-assessment of uncanniness (uncanny feelings) from robot and human stimuli were rated by the participants with a 1–9-point scale where 1 point was for normal/neutral/trustful feelings and 9 points were given for the uncanny/eerie/negative feelings. Before the experiment, the meaning of the “Uncanny” and aim of the measurement was explained clearly to participants with examples involving unpleasant, strange and eerie concepts. Experiment was conducted with Turkish participants and Turkish translations of the words were also placed in the rating screen. All four robot and human stimuli were presented twice and rated by the participants. This behavioral episode took about 2 min (Figure 2B).

### fNIRS Data Acquisition

**Figure 3:**
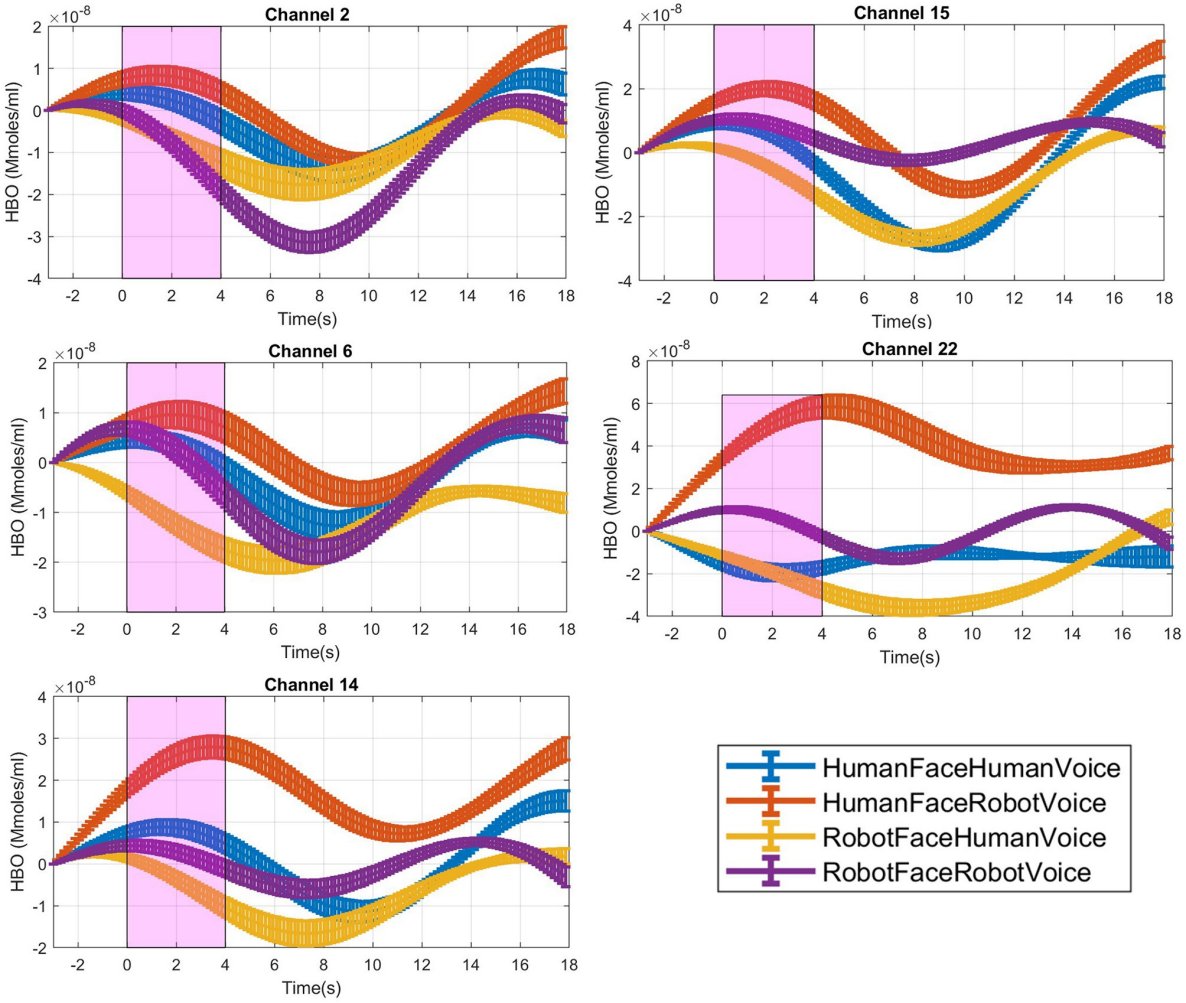
Time traces of block averaged Δ[HbO] signals of each audiovisual stimuli in representative channels, averaged across all subjects. Δ[HbO] resembles the relative concentration change of HbO in micromoles at probed brain tissue. Each time trace indicates block average of HbO responses detected during each specific audio-visual stimulus from the same channel of all participants. Pink shaded area represents the audio-visual stimulus interval. Error bars indicate the standard error of the mean signal across participants.

A NIRSport functional near infrared spectroscopy system (NIRSport, NIRx Medical Technologies LLC, Berlin, Germany, https://nirx.net/) was used to measure prefrontal cortical activation during presentation of stimuli. The system involves 22 channels which consist of 8 light sources (emitting near-infrared light at 760 and 850 nm) and 7 detectors. The combinations of light source-detector pairs with a 3 cm distance were accepted as channels. All channels collected hemodynamic data from the frontal cortex region (Figure 3). Concentration changes of HbO and HbR were calculated with the modified Beer-Lambert law (Jöbsis, 1977). The fNIRS signals had a 7.8125 Hz sampling frequency. Profile of probe sensitivity was calculated by the Atlas Viewer toolbox et al., of Homer2 Software to ensure that all channels collect data from the first few millimeters of the frontal cortex (Huppert et al., 2009; Aasted et al., 2015; Mutlu et al., 2020). AtlasViewer toolbox enables mapping of each channel's photon propagation density onto cerebral and non-cerebral layers beneath the corresponding source-detector pair. The optical sensitivity profile of each channel was first coregistered on a standard brain template with a 10/5 global EEG electrode system. The channel locations were also coregistered onto a standard brain in MNI space using the NIRS_SPM toolbox (Jang et al., 2009; Ye et al., 2009; Vos et al., 2012). The percentage of Brodmann areas covered by the propagating photons of each channel was computed with the spatial registration toolbox of NIRS_SPM by use of the Rorden's brain atlas (Rorden and Brett, 2000). The Brodmann areas covered by each channel of the utilized forehead probe are listed in a previous work of our research group (Mutlu et al., 2020).

### Data Analysis

#### Analysis of Behavioral Data

Twenty-nine participants rated the audio-visual human and robot videos between normal and uncanny feelings. Average uncanniness scores for each of the 4 audio-visual stimuli were calculated. A 2 x 2 [Face (Human, Robot) x Voice (Human, Robot)] repeated measures ANOVA were performed on uncanniness scores.

#### fNIRS Data Preprocessing and Feature Selection

Data preprocessing of fNIRS signals involved several steps. Initial visual inspection of data was performed with nirsLAB (NIRX Medical Technologies, Berlin, Germany, https://nirx.net/nirslab-1) toolbox. Detailed analysis and preprocessing steps were carried out with HOMER2 scripts and customized MATLAB scripts (Mathworks, Natick, MA, USA, Stearns and Hush, 2016) scripts. First, channels with poor signal quality were eliminated using enPruneChannels.m function of the HOMER2 toolbox (Cooper et al., 2012). Raw light intensity data were transformed to optical density (OD) data by using hmrIntensity2OD.m function. Motion artifacts in channel-wise time series were detected using HOMER2-hmrMotionArtifact.m function with the following parameters: Motion = 0.5, tMask = 1, STDEVthresh = 10, and AMPthresh = 1. Principal component analysis was employed to remove motion artifacts *via* the HOMER2-hmrMotionCorrectPCA.m function (nSV parameter = 0.8). After motion correction, OD data were filtered with a Butterworth bandpass filter which had a high frequency cut-off at 0.005 Hz and a low frequency cut-off at 0.08 Hz. As the final data preprocessing step; HbO and HbR concentrations were calculated from filtered OD data based on the Modified Beer-Lambert law with HOMER2-hmrOD2Conc.m function (Baker et al., 2014).

Only HbO data were included in the analysis since HbO is considered as a more reliable and well-grounded marker of cortical hemodynamic activation (Watanabe et al., 2002; Scholkmann et al., 2014; Dravida et al., 2017). Hemodynamic signals from fNIRS recordings contain our signals of interest which are the neuronally induced hemodynamic changes but these neuronally induced effects are intermixed with cerebral and extracerebral physiological effects such as heart beat, respiration and Mayer waves. These systemic physiological effects may cause false negative and false positive activation patterns if they are not properly eliminated (Yücel et al., 2016). Assuming that the systemic physiological activity is common across all channels, a principal component analysis was applied to whole channel data. Top principal components which explained 75 % of the covariance of all channel data were accepted as regressors modeling the common noise effect and they were linearly regressed out from time series of each channel separately to isolate and extract the neurally induced hemodynamic effects (Mutlu et al., 2020).

#### fNIRS Data Analysis

For each channel's preprocessed HbO signal, 18-s-long block segments were extracted, which included each 4-s-long single trial with a 3 s pre-stimulus baseline interval and an 11 s post stimulus interval. Each trial segment was detrended to remove the linear trend from the data and was classified into one of the four categories of the audio-visual stimuli trial types. The mean HbO signal for each condition and subject was computed by averaging across time series of all trial blocks (*n* = 10) belonging to each stimulus type. Block averaging procedure was performed for each subject and channel's HbO signal data separately. Hence, a single-block averaged time-series HbO signal data was calculated for each condition and channel for each subject.

For each stimulus trial, a hemodynamic effect size metric named Cohen's D was computed by subtracting the mean of the signal in the [0–3] s pre-stimulus time range before the stimulus onset from the mean of the signal in the [1–4] s duration after the stimulus onset (Balconi and Molteni, 2016). Stimulus induced amplitude change with respect to the baseline was normalized by dividing the difference between the mean baseline amplitude and task mean amplitude with the standard deviation of the [1–3] s pre-stimulus baseline (Balconi et al., 2015; Balconi and Vanutelli, 2016, 2017; Mutlu et al., 2020), (Figure 3).

#### Statistical Analysis of HbO Signal Data

A 2 x 2 repeated measures ANOVA [factors: face (human, robot), voice (human, robot)] was employed on Cohen's D metric of each channel separately to localize statistically significant differences in HbO signal activation at the group level across different stimuli types. *Post-hoc* analyses were performed with a Bonferroni correction procedure. Pearson's correlation between Cohen's D metrics and all participant's behavioral rating scores were calculated and illustrated in scatter plots (**Figure 6**). All statistical analyses were executed with JASP software (University of Amsterdam, Netherlands), (Love et al., 2019).

## Results

### Behavioral Experiments

**Figure 4:**
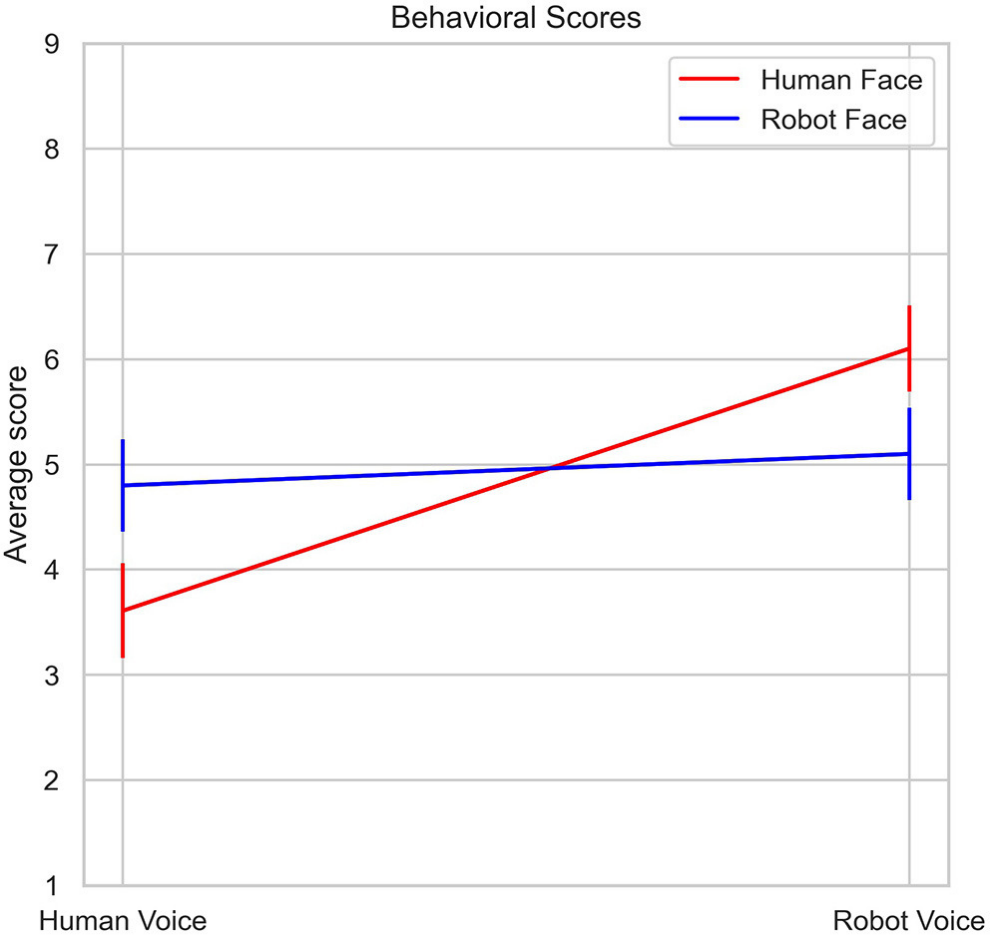
Changes in average Uncanniness score with respect to voice type and face type.

Mean uncanniness scores for all stimuli types are listed in Table 1 and illustrated in Figure 4. Analysis of 2 (Face: Human, Robot) x 2 (Voice: Human, Robot) mixed ANOVA on stimuli ratings showed a strong main effect of voice [F (1, 29) = 15.5, *p* < 0.001, η2p = 0.35], but the main effect of face was not significant [F (1, 29) = 0.1, *p* = 0.76, η2p = 0.003]. There was also an interaction between face and voice stimuli [F (1, 29) = 8.47, *p* = 0.007, η2p = 0.23]. Human face - robot voice stimuli elicited significantly higher uncanniness scores than the human face - human voice stimuli (t = −4.84, *p* < 0.001). Robot face - robot voice stimuli also elicited significantly higher uncanniness scores than the human face-human voice stimuli (t = −3.13, *p* < 0.017). Bonferroni corrections were applied for the analysis.

**Table 1:** Average uncanniness scores of audio-visual stimuli.

**Stimuli type**	**Average score**
Human face-human voice	3.61 ± 0.45
Human face-robot voice	6.12 ± 0.41
Robot face-human voice	4.84 ± 0.44
Robot face-robot voice	5.18 ± 0.44

### fNIRS Experimental Results

**Figure 5:**
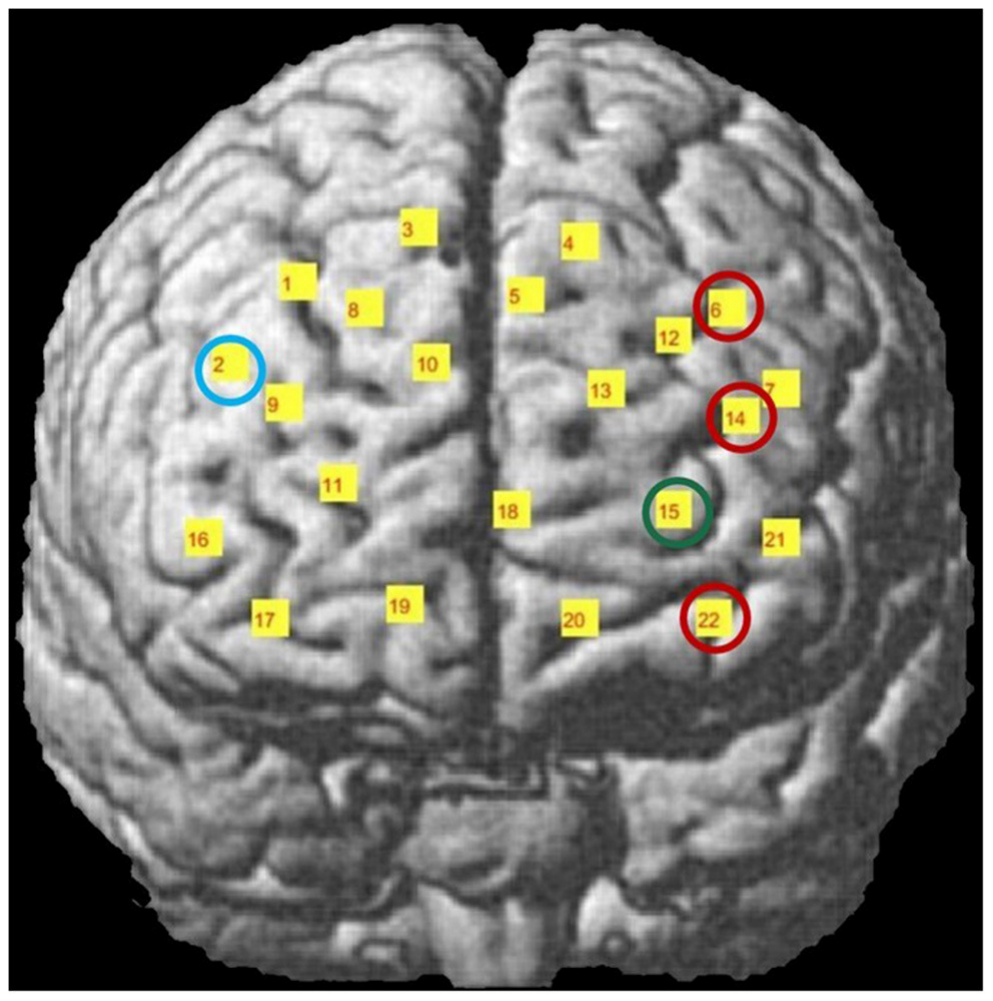
Channel locations of fNIRS probe mapped onto a standard brain template in MNI space. Channels with significant hemodynamic activation are illustrated with colored circles. (Red circle: Human Face > Robot Face, Green circle: Robot Voice < Human Voice, Blue circle: Human Face Robot Voice > Robot Face Robot Voice). Adapted from Mutlu et al. (2020).

Differences in Cohen's D metric of all subjects across different trial types were statistically analyzed with a 2^*^2 ANOVA design for each channel separately. A main effect of face was observed in Channels 6, 14, 22 while a main effect of voice was observed in Channel 15 and a face-voice interaction effect was observed in Channel 2 which are schematically demonstrated in Figure 5. Main effect of the face was observed in 2 channels located in the left dorsolateral prefrontal cortex (DLPFC) and one channel located in the left orbitofrontal cortex (OFC) (red circles). Main effect of voice was observed only in the left OFC (green circle) and the face-voice interaction effect was observed in one channel located in the right DLPFC (blue circle), (Figure 5).

**Figure 6:**
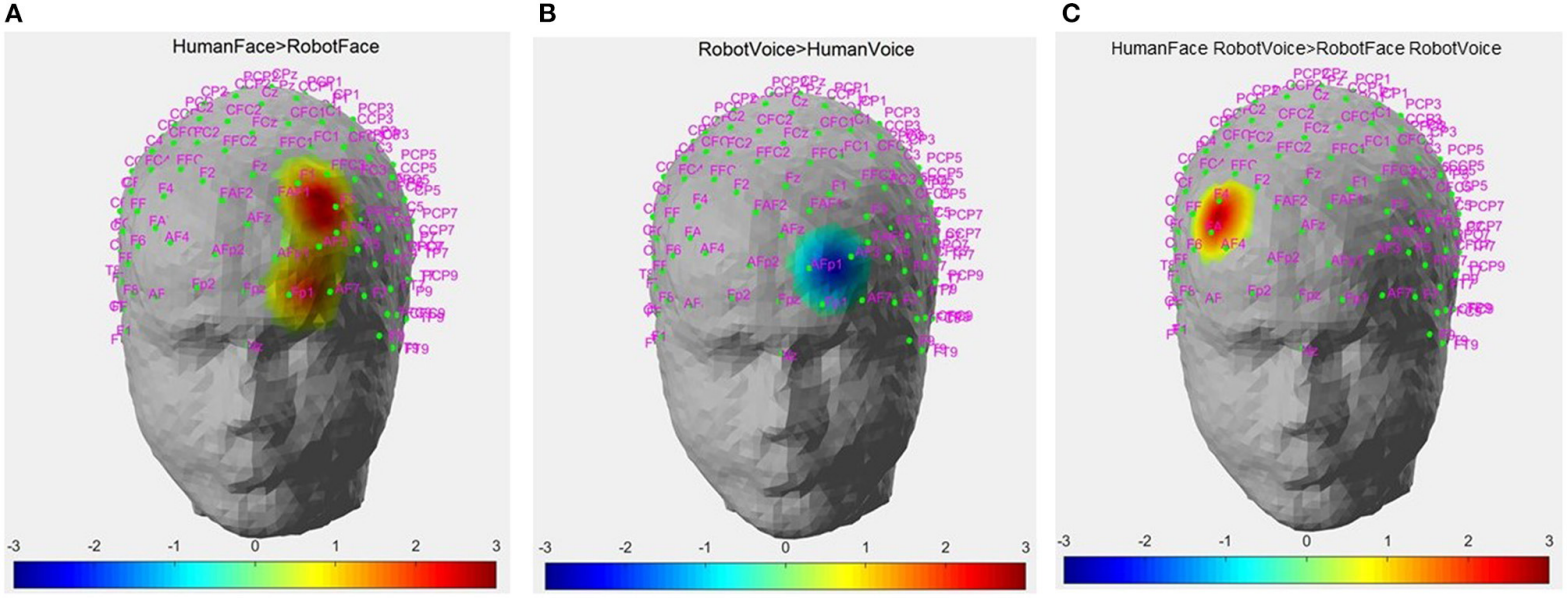
Hemodynamic activation maps obtained during different contrasts between audiovisual stimuli. **(A)** Human Face > Robot Face contrast, **(B)** Robot Voice < Human Voice contrast, **(C)** Face-voice interaction: Human Face-Robot Voice > Robot Face-Robot Voice contrast. T scores of channels showing statistically significant activation (*p* < 0.05) are mapped on to the standard head model map. Color bar represents threshold t statistic scores.

*Post-hoc* analyses indicated that presentation of human face stimuli elicited significantly higher hemodynamic responses at Channel 6, Channel 14 and Channel 22, when compared to the hemodynamic responses obtained during presentation of robot face stimuli [for Channel 6: F (1, 29) =7.3, t = 2.7, *p* = 0.012, for Channel 14: F (1, 29) = 4.2, t = 2.05, *p* = 0.05, for Channel 22: F (1, 29) = 7.24, t = 2.69, *p* = 0.012], (Figure 6A). Significantly lower hemodynamic response to the presentation of robot voice was detected at Channel 15 when compared to presentation of human voice [F (1, 29) = 5.62, t = −2.37, *p* = 0.025), (Figure 6B). Significantly higher hemodynamic responses to human face-robot voice stimuli were detected at Channel 2 [F (1, 29) = 8.33, t = 2.87, *p* = 0.045] when compared to robot face robot-voice stimuli (Figure 6C; Table 2). Bonferroni corrections were applied for the analysis.

**Table 2:** *Post-hoc* comparisons of Cohen's D metric obtained during different types of stimuli at the significant channels.

**Channel**	**Stimuli**	**Mean diffference (CI:%95)**	**F (1, 29)**	**t**	***P*** **-value**
2	Human face-robot voice> Robot face-robot voice	7.49	8.33	2.78	0.045
6	Human face>robot face	3.42	7.3	2.7	0.012
14	Human face>robot face	2.1	4.2	2.05	0.05
15	Robot voice < human voice	−2.72	5.62	−2.37	0.025
22	Human face> robot face	3.27	7.24	2.69	0.012

**Figure 7:**
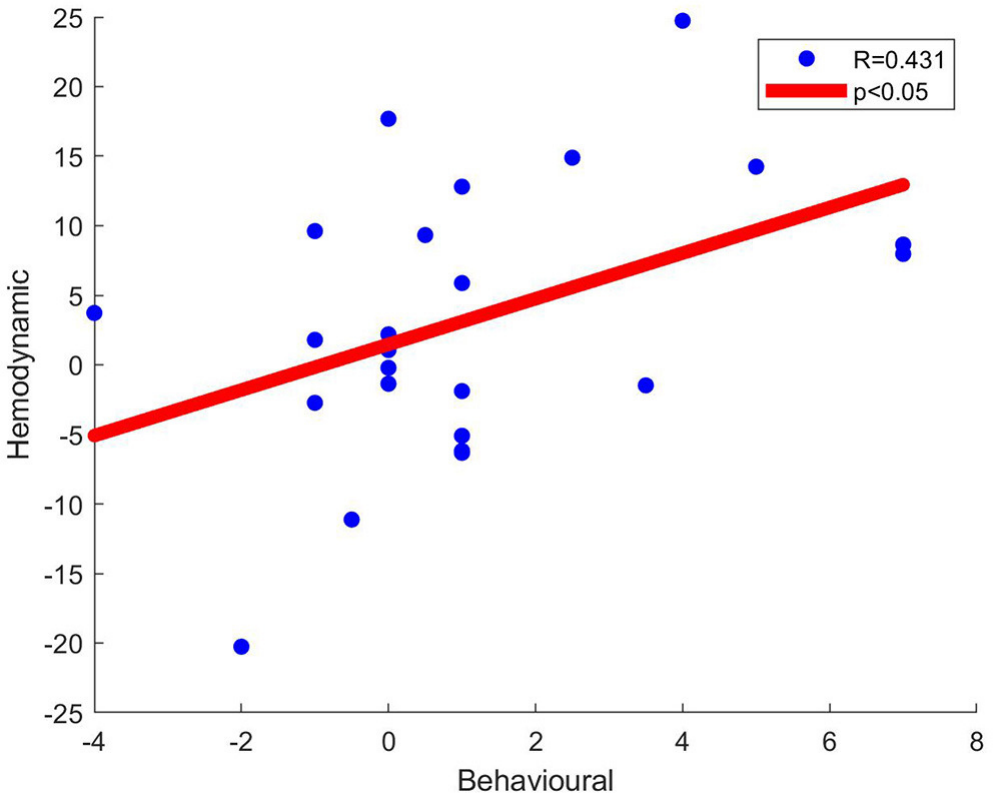
Scatter plots of behavioral data (i.e., Uncanniness Rating) and Cohen's D metrics computed for Human Face - Robot Voice > Robot Face - Robot Voice contrast. Each dot represents a comparison of changes in behavioral score and hemodynamic parameter of channel 22 for each subject. The two variables were found to be linearly correlated (*p* < 0.05).

Pearson's correlation coefficients were computed between behavioral ratings of the stimuli and Cohen's D metric of significantly active channels. At Channel 22, significant moderate correlation between behavioral ratings and Cohen's D hemodynamic activity metric was detected for the Human Face - Robot Voice > Robot Face - Robot Voice contrast (R = 0.431, *n* = 24 and *p* < 0.05), (Figure 7).

## Discussion

In this study, we aimed to investigate the spatiotemporal features of cortical hemodynamic responses of healthy adults during perception of various types of audio-visual human and robot video stimuli. We aimed to observe correlations between behavioral responses (i.e., uncanniness score) and magnitude of hemodynamic activity in significantly activated PFC regions. The ultimate goal was to interpret the differences and similarities between neural correlates of emotional processing of human and robot faces and to explore whether the uncanny feelings elicited a proportional hemodynamic effect that could be quantified with fNIRS recordings.

Distinct neural responses to human and robot stimuli were detected at the DLPFC and OFC regions in the present study. Two channels which are located in the left DLPFC, one channel at right DLPFC and two channels in left OFC regions showed significant hemodynamic activity during processing of different human and robot stimuli. Significantly active channels relevantly localized around the lateral parts of the PFC. The fact that our robot and human stimuli elicit significant hemodynamic responses at the DLPFC regions may be considered as a verification that our experimental design induced the desired hemodynamic contrast in alignment with current literature and could be used to test our hypotheses.

Our results demonstrate that the hemodynamic response to human face stimuli is statistically significantly higher than the responses to robot face stimuli in Channels 6 and 14 which are located in the left DLPFC region (Figure 6A). Human face is one of the main sources of social cognition as a strong representation of identity and induces emotional states with facial mimicry (Leopold and Rhodes, 2010). Higher hemodynamic response to stimuli involving human faces may be linked to the primary emotion regulation function of the DLPFC (Kaller et al., 2011). DLPFC regions have been shown to elicit significant cortical activation in response to human face stimuli in previous studies conducted with fMRI. Lower degrees of DLPFC activation to happy faces was observed in depression patients when compared to healthy controls (Manelis et al., 2019). A recent study by Kelley et al. explored differences in hemodynamic activity during a paradigm involving a human to human eye contact and human to robot eye contact and found that human to human eye contact elicited greater DLPFC activity (Kelley et al., 2021).

Previous fMRI studies demonstrated that human and robot stimuli elicited neural responses in specific brain regions such as fusiform gyrus and temporo-parietal junction (Özdem et al., 2016; Hogenhuis, 2021; Kelley et al., 2021). PFC, the most complex and executive part of the human brain, also has a role in perception and emotional processing steps in human-robot interactions (HRI). Ventromedial parts of the prefrontal cortex (vmPFC) are addressed with social cognition capabilities such as theory of mind and facial emotion cognition (Hiser and Koenigs, 2018). Hence, the role of vmPFC in social cognition can be expanded to HRI as well. An experimental human-robot and human-human interaction paradigm elicited increased activity in vmPFC regions in a study performed by Wang and Quadflieg (2014). DLPFC and other PFC regions have indirect roles in emotion regulation and are involved primarily in regulation, integration and processing of executive functions such as cognitive control and secondarily abstract reasoning processes (Kaller et al., 2011). DLPFC lesions caused impaired auditory attention (Bidet-Caulet et al., 2015). Lateral OFC (IOFC) was also found sensitive to facial mimicry and has been shown to play a role in generating emotional response to human faces (Howard et al., 2015; Dixon et al., 2017). However, a recent fNIRS study presented that artificially designed faces and facial expressions elicited less hemodynamic response than real human faces (Zhao et al., 2020).

We used neutral human and robot faces with short greeting sentences as stimuli in our study. Human face is the primary instrument during social interactions and consists of a very intense and multidimensional emotional context which includes facial expressions, identical clues and personal traits (Jack and Schyns, 2015). Neutral faces are accepted as standard types of facial expressions and matched with objective emotions in a well-known study on emotions by Ekman (Ekman, 2017). However; neutral faces may be perceived with negative or positive emotions depending on the experimental context (Lee et al., 2008; Said et al., 2009). PFC and subregions, our research zone, have high ordered capabilities in emotion regulation and are sensitive to human faces (Wolf et al., 2014). In addition to neuroimaging of PFC regions, we also asked participants if human and robot stimuli create uncanny or normal feelings during the experiment. Emotional response to faces were evaluated with rating scores and increased PFC activity to human face stimuli was detected in an fMRi neuroimaging study (Morita et al., 2008). Even short-term greetings have been reported to induce positive emotional response in observers during human-robot interaction in a recent study by Fischer et al. (2019). Design and the level of human likeness of the robot faces also changes emotional response in observers (Appel et al., 2020).

At first glance, consistent localizations of channels with significant changes in hemodynamic response in the DLPFC region may be linked with emotional regulation and processing of human faces. Based on previous experimental studies, human and/or robot faces with a greeting message as presented in the current study may be considered as emotional stimuli (Morita et al., 2008; Fischer et al., 2019). We propose that higher hemodynamic responses in DLPFC and OFC regions during observation of human face stimuli might be modulated by the emotions induced by the faces.

Another PFC region where stimuli involving human faces induce significantly higher hemodynamic response than that of robot faces corresponds to Channel 22 which is located in the OFC region. OFC regions are also sensitive to processing of human faces and face specific activation changes were observed in OFC during face selection tasks (Barat et al., 2018). OFC has also been shown to elicit activation in the infant brain during parenteral interaction which involves face and voice stimuli (Parsons et al., 2013).

Significantly lower hemodynamic responses to robot voice were observed in a left OFC channel (Channel 15) when compared to human voice stimuli (Figure 6B). Several fNIRS neuroimaging studies indicated that conversation with robots creates complex PFC activity (Kawaguchi et al., 2011; Strait et al., 2014). However, unlike stimuli involving conversation, the robot voice stimuli used in the present study contains a few words and it is unidirectional. Our results provide complementary information to previous literature as we demonstrate that short lasting robot voices also elicit decreased hemodynamic activity in the OFC when compared to human voice stimuli. This finding supports OFC's differential sensitivity to processing human and robot voices even during a short-term trial. Robot voices may create avoidance feelings in humans which may manifest decreased hemodynamic response in OFC regions. Indeed, OFC was found to be sensitive to human voices and impairment of OFC regions resulted in disruption of the voice identification process in the human brain (Hornak et al., 2003; Parsons et al., 2013). Lateral OFC was also found to be sensitive to facial mimicry and plays a significant role in generating emotional response to human faces (Wang and Quadflieg, 2014; Bidet-Caulet et al., 2015). Similar to our findings, a recent fNIRS study presented that artificially designed faces and facial expressions elicited less hemodynamic response than real human faces (Zhao et al., 2020).

In our study, participants rated human and robot stimuli immediately after the fNIRS recording and behavioral results in the form of uncanniness score were also evaluated. Human face-robot voice paired stimuli were rated as inducing the most uncanny feeling (Figure 5), followed by robot face-robot voice and robot face-human voice stimuli (Table 1). Human face-human voice stimuli were rated as most normal. There is a clear contrast between the results where incongruent human face-robot voice stimuli are perceived as the most uncanny, and congruent human face-human voice stimuli are perceived as the most normal. Mismatch between face and voice creates discomfort and disharmony feelings in humans. According to anthropological and evolutionary theories; cognitive dissonance may be a plausible reason for disturbance feelings from mismatched situations (Laue, 2017).

In our experimental paradigm; we primarily used human and robot faces and voices as emotional stimuli. Face perception is a special step of social perception and emotions are constructed from perceptions and evaluations during social interactions (Scherer and Moors, 2019). In addition to congruent pairs, our experimental paradigm also included incongruent face and voice pairs of humans and robots to explore responses to conflicting stimuli in the PFC. Face-voice mismatch effect violates categorical boundaries about robot and human definitions in our perception, as previous studies openly presented (Mitchell, 2011; Meah and Moore, 2014; Yorgancigil et al., 2021). Conflicting stimuli tasks can activate left DLPFC regions which was revealed in an fMRI study (Wittfoth et al., 2009). Findings from another fMRI study showed that medial and lateral PFC regions have also been activated during a Stroop task paradigm which involved visual stimuli conflict (Egner and Hirsch, 2005). With a broader view, perceptual and cognitive effects of robots can be addressed in PFC regions in the human brain. In alignment with the increasing uncanniness score with face - voice mismatch effect (Figure 4), incongruent human face - robot voice stimuli elicited significantly higher hemodynamic response than congruent robot face - robot voice stimuli in a DLPFC channel (Channel 2, Figure 6C). In a previous fMRI study, face - voice mismatch also resulted in significant alterations in the neural activity of the inferior frontal gyrus, which is a neighboring region of the PFC (Uno et al., 2015).

Another main finding of our study is the significant correlation between hemodynamic activity of a left OFC channel and the extent of uncanny feelings (Figure 7). At Channel 22, human face - robot voice > robot face - robot voice hemodynamic contrast was accompanied with a significant correlation between behavioral (i.e., uncanniness score) and hemodynamic differences depicting the same contrast. The difference in uncanniness score between human face-robot voice stimuli and robot face-robot voice is positive in all subjects and as this mismatch increases, the difference in the hemodynamic response obtained during processing these two types of stimuli also increases. Channel 22 also shows statistically significantly higher hemodynamic response to human face stimuli when compared to robot face stimuli (Figure 6A). Human face perception process has specific neural regions in the human brain and top-down modalities are employed during face perception (Ban et al., 2004). Top-down modalities govern face perception by integrating information from various parts of the brain (Oruc et al., 2019). Consequently, we propose that behavioral and hemodynamic correlation of increased response to the human face may be a sign of integrated perception of the human face at the OFC regions.

### Limitations of the Study and Recommendations for Future Work

Our study has several limitations in terms of experimental design and the capability of the utilized neuroimaging technology. The experimental design consists of videos involving a mix of human and robot face and audio stimuli for the purpose of evaluating the hemodynamic processing of human - robot interactions. Our robot and human audio-visual stimuli were presented from a computer screen which might induce additional neuronal processing in the PFC and introduce some confounding effects to the spatiotemporal patterns of hemodynamic activity. While the majority of cognitive experiments conducted with fNIRS and fMRI involve presentation of audio-visual stimuli from digital interfaces, we should still highlight the fact that future work should involve repeating the same experiments with real humans and robot models in a real-world setting to exclude the potential hemodynamic confounding effects introduced by visual processing of the digital interface. In addition to robot face representation in a video format, robot models allow interaction with all parts of the robot body and tactile sensation. Our results are limited with interpretation of the hemodynamic activity of the PFC due to the limited channel number of our fNIRS device. Human and robot face stimuli elicit PFC activity due to induction of the emotion regulation and social cognition capabilities, however, cerebral cortical regions such as temporoparietal junction and fusiform gyrus are also very well-known to be addressed during human-robot interactions. We will aim at concurrent investigation of these cerebral regions by integrating fMRI measurements to our protocols for future work. Whole brain imaging promises better spatial resolution for analysis of hemodynamic responses induced by human-robot interaction in the cerebral cortex. We also propose that more advanced experimental paradigms which execute real human and robot model conditions may provide richer emotional and behavioral clues which can be detected with fNIRS in future work.

### Novelty of the Findings

Current study presents the following novelties with respect to the current literature. To date, there have been no studies that aimed to explore the neurophysiological underpinning of the face and voice congruence. We examined the processing of a face-voice mismatch condition during presentation of robot and human stimuli with fNIRS modality, which is a relatively novel functional imaging modality that enables real time quantification of brain hemodynamic responses to presented stimuli. Here, we conducted an exploratory study of how human and robot faces are perceived and emotionally processed in the PFC by computing the hemodynamic correlates of neural activation during both types of stimuli using the fNIRS method. PFC is one of the prominent brain areas where the high-level cognitive processes such as emotion regulation, social cognition and identity of self are located (Hiser and Koenigs, 2018). Our study demonstrates the potential and feasibility of fNIRS as a convenient and practical neuroimaging method for quantification of human-robot interactions in natural environments of participants. fNIRS also provides robust presentation of task-related hemodynamic activity in the human brain with minimized external noise, low operating costs, quick set-up time and calibration (Balconi and Molteni, 2016).

## Conclusion

In support of previous literature, DLPFC and OFC regions were found to be sensitive to processing of stimuli consisting of robot and human faces in the present study. Our study provides an insight to the neural mapping of human and robot conditions in the PFC and contributes to elucidation of the neural processing of human and robot stimuli in the PFC in naturalistic conditions. We believe inclusion of a wider population of participants and more sophisticated experimental conditions will pave the way to finding hemodynamic markers of perceptual classification of human and robot faces in the human brain.

## Data Availability Statement

The raw data supporting the conclusions of this article will be made available by the authors, without undue reservation.

## Ethics Statement

The studies involving human participants were reviewed and approved by Non-interventional Research Ethics Board of Medipol University, Istanbul, Turkey. The patients/participants provided their written informed consent to participate in this study. Written informed consent was obtained from the individual(s) for the publication of any potentially identifiable images or data included in this article.

## Author Contributions

EY, FY, BU, and SE designed the experiments. EY and SE carried out the experiments, analyzed the data, wrote the manuscript, and prepared the figures. All authors reviewed the manuscript, contributed to the article, and approved the submitted version.

## Conflict of Interest

The authors declare that the research was conducted in the absence of any commercial or financial relationships that could be construed as a potential conflict of interest.

## Publisher's Note

All claims expressed in this article are solely those of the authors and do not necessarily represent those of their affiliated organizations, or those of the publisher, the editors and the reviewers. Any product that may be evaluated in this article, or claim that may be made by its manufacturer, is not guaranteed or endorsed by the publisher.

## References

[B1] Aasted C. M. Yücel M. A. Cooper R. J. Dubb J. Tsuzuki D. Becerra L.. (2015). Anatomical guidance for functional near-infrared spectroscopy: atlasviewer tutorial. Neurophotonics 2, 020801. 10.1117/1.NPh.2.2.020801 26157991 PMC4478785

[B2] Appel M. Izydorczyk D. Weber S. Mara M. Lischetzke T. (2020). The uncanny of mind in a machine: humanoid robots as tools, agents, and experiencers. Comput. Human Behav. 102, 274–286. 10.1016/j.chb.2019.07.031

[B3] Baker W. B. Parthasarathy A. B. Busch D. R. Mesquita R. C. Greenberg J. H. Yodh A. G. (2014). Modified Beer-Lambert law for blood flow. Biomed. Opt. Express 5, 4053–4075. 10.1364/BOE.5.004053 25426330 PMC4242038

[B4] Balconi M. Grippa E. Vanutelli M. E. (2015). What hemodynamic (fNIRS), electrophysiological (EEG) and autonomic integrated measures can tell us about emotional processing. Brain Cogn. 95, 67–76. 10.1016/j.bandc.2015.02.001 25721430

[B5] Balconi M. Molteni E. (2016). Past and future of near-infrared spectroscopy in studies of emotion and social neuroscience. J. Cogn. Psychol. 28, 129–146. 10.1080/20445911.2015.110291927812329

[B6] Balconi M. Vanutelli M. (2016). Hemodynamic (fNIRS) and EEG (N200) correlates of emotional inter-species interactions modulated by visual and auditory stimulation. Sci. Rep. 23083. 10.1038/srep23083 26976052 PMC4791677

[B7] Balconi M. Vanutelli M. E. (2017). Empathy in negative and positive interpersonal interactions. what is the relationship between central (EEG, fNIRS) and peripheral (autonomic) neurophysiological responses? Adv. Cogn. Psychol. 13, 105–120. 10.5709/acp-0211-0 28450977 PMC5402676

[B8] Ban S. W. Lee M. Yang H. S. (2004). A face detection using biologically motivated bottom-up saliency map model and top-down perception model. Neurocomputing 56, 475–480. 10.1016/j.neucom.2003.10.003

[B9] Barat E. Wirth S. Duhamel J.-R. (2018). Face cells in orbitofrontal cortex represent social categories. Proc. Natl. Acad. Sci. 115, E11158–E11167. 10.1073/pnas.1806165115 30397122 PMC6255179

[B10] Bidet-Caulet A. Buchanan K. G. Viswanath H. Black J. Scabini D. Bonnet-Brilhault F.. (2015). Impaired facilitatory mechanisms of auditory attention after damage of the lateral prefrontal cortex. Cereb. Cortex 25, 4126–4134. 10.1093/cercor/bhu131 24925773 PMC4626830

[B11] Breazeal C. Dautenhahn K. Kanda T. (2016). Social robotics, in Springer Handbook of Robotics. (Berlin; Heidelberg: Springer-Verlag), 1935–1971.

[B12] Cooper R. Selb J. Gagnon L. Phillip D. Schytz H. W. Iversen H. K.. (2012). A systematic comparison of motion artifact correction techniques for functional near-infrared spectroscopy. Front. Neurosci. 147. 10.3389/FNINS.2012.00147 23087603 PMC3468891

[B13] Dixon M. L. Thiruchselvam R. Todd R. Christoff K. (2017). Emotion and the prefrontal cortex: an integrative review. Psychol. Bull. 143, 1033–1081. 10.1037/bul0000096 28616997

[B14] Dravida S. Noah J. A. Zhang X. Hirsch J. (2017). Comparison of oxyhemoglobin and deoxyhemoglobin signal reliability with and without global mean removal for digit manipulation motor tasks. Neurophotonics 5:1. 10.1117/1.nph.5.1.011006 28924566 PMC5597778

[B15] Egner T. Hirsch J. (2005). The neural correlates and functional integration of cognitive control in a stroop task. NeuroImage 24, 539–547. 10.1016/j.neuroimage.2004.09.007 15627596

[B16] Ekman P. (2017). Facial expressions of emotion: new findings, new questions. Psychol. Sci. 3, 34–38. 10.1111/j.1467-9280.1992.tb00253.x

[B17] Erdogan S. B. Yücel M. A. Akin A. (2014). Analysis of task-evoked systemic interference in fNIRS measurements: insights from fMRI. NeuroImage 87, 490–504. 10.1016/j.neuroimage.2013.10.024 24148922

[B18] Fischer K. Jung M. Jensen L. C. Aus Der Wieschen M. V. (2019). Emotion expression in HRI - when and why, in ACM/IEEE International Conference on Human-Robot Interaction (Daegu), 29–38.

[B19] Forbes C. E. Grafman J. (2010). The role of the human prefrontal cortex in social cognition and moral judgment. Annu. Rev. Neurosci. 33, 299–324. 10.1146/annurev-neuro-060909-153230 20350167

[B20] Henschel A. Hortensius R. Cross E. S. (2020). Social cognition in the age of human–robot interaction. Trends Neurosci. 43, 373–384. 10.1016/j.tins.2020.03.013 32362399

[B21] Hiser J. Koenigs M. (2018). The multifaceted role of the ventromedial prefrontal cortex in emotion, decision making, social cognition, and psychopathology. Biol. Psychiatry 83, 638–647. 10.1016/j.biopsych.2017.10.030 29275839 PMC5862740

[B22] Hogenhuis A. L. M. P. (2021). The Difference in Functional Connectivity During Human-Human Interaction and Human-Robot Interaction. Available online at: http://dspace.library.uu.nl/handle/1874/402819 (accessed July 04, 2022).

[B23] Hornak J. Bramham J. Rolls E. T. Morris R. G. O'Doherty J. Bullock P. R.. (2003). Changes in emotion after circumscribed surgical lesions of the orbitofrontal and cingulate cortices. Brain 126, 1691–1712. 10.1093/brain/awg168 12805109

[B24] Howard J. D. Gottfried J. A. Tobler P. N. Kahnt T. (2015). Identity-specific coding of future rewards in the human orbitofrontal cortex. Proc. Natl. Acad. Sci. 112, 5195–5200. 10.1073/pnas.1503550112 25848032 PMC4413264

[B25] Huppert T. J. Diamond S. G. Franceschini M. A. Boas D. A. (2009). HomER: a review of time-series analysis methods for near-infrared spectroscopy of the brain. App. Optics 48, D280–D298. 10.1364/AO.48.00D280 19340120 PMC2761652

[B26] Hwang E. Lee J. (2021). Attention guidance technique using visual subliminal cues and its application on videos; attention guidance technique using visual subliminal cues and its application on videos, in ACM International Conference on Interactive Media Experiences (New York City, NY).

[B27] Jack R. E. Schyns P. G. (2015). The human face as a dynamic tool for social communication. Curr. Biol. 25, R621–R634. 10.1016/j.cub.2015.05.052 26196493

[B28] Jang K.-E. Tak S. Jung J. Jang J. Jeong Y. (2009). Wavelet minimum description length detrending for near-infrared spectroscopy. J. Biomed. Opt. 14, 034004. 10.1117/1.3127204 19566297

[B29] Jöbsis F. F. (1977). Noninvasive, infrared monitoring of cerebral and myocardial oxygen sufficiency and circulatory parameters. Science 198, 1264–1267. 10.1126/science.929199 929199

[B30] Kaller C. P. Rahm B. Spreer J. Weiller C. Unterrainer J. M. (2011). Dissociable contributions of left and right dorsolateral prefrontal cortex in planning. Cereb. Cortex. 21, 307–317. 10.1093/cercor/bhq096 20522540

[B31] Kawaguchi Y. Wada K. Okamoto M. Tsujii T. Shibata T. Sakatani K. (2011). Investigation of brain activity during interaction with seal robot by fNIRS, in Proceedings - IEEE International Workshop on Robot and Human Interactive Communication (Georgia, GA), 308–313.

[B32] Kelley M. S. Noah J. A. Zhang X. Scassellati B. Hirsch J. (2021). Comparison of human social brain activity during eye-contact with another human and a humanoid robot. Front. Robot. AI 7, 599581. 10.3389/frobt.2020.599581 33585574 PMC7879449

[B33] Laue C. (2017). Familiar and strange: Gender, sex, and love in the uncanny valley. Multi. Technol. Interaction. 1:2. 10.3390/mti1010002

[B34] Lazarus R. S. (1991). Emotion and Adaptation. Available online at: https://philpapers.org/rec/SLAEAA (accessed July 04, 2022).

[B35] Lee E. Kang J. I. Park I. H. Kim J. J. An S. K. (2008). Is a neutral face really evaluated as being emotionally neutral? Psychiatry Res. 157, 77–85. 10.1016/j.psychres.2007.02.005 17804083

[B36] Leopold D. A. Rhodes G. (2010). A comparative view of face perception. J. Comp. Psychol. 124, 233–251. 10.1037/a0019460 20695655 PMC2998394

[B37] Love J. Selker R. Marsman M. Jamil T. Dropmann D. Verhagen J.. (2019). JASP: Graphical statistical software for common statistical designs. J. Stat. Softw. 88, 1–17. 10.18637/jss.v088.i02

[B38] Manelis A. Huppert T. J. Rodgers E. Swartz H. A. Phillips M. L. (2019). The role of the right prefrontal cortex in recognition of facial emotional expressions in depressed individuals: fNIRS study. J. Affect. Disord. 258, 151–158. 10.1016/j.jad.2019.08.006 31404763 PMC6710146

[B39] Meah L. F. S. Moore R. K. (2014). The uncanny valley: A focus on misaligned cues, in Social Robotics. ICSR 2014. Lecture Notes in Computer Science, Vol. 8755, eds Beetz M. Johnston B. Williams M. A. (Cham: Springer). 10.1007/978-3-319-11973-1_26

[B40] Mitchell D. G. V. (2011). The nexus between decision making and emotion regulation: a review of convergent neurocognitive substrates. Behav. Brain Res. 217, 215–231. 10.1016/j.bbr.2010.10.030 21055420

[B41] Morita T. Itakura S. Saito D. N. Nakashita S. Harada T. Kochiyama T.. (2008). The role of the right prefrontal cortex in self-evaluation of the face: a functional magnetic resonance imaging study. J. Cogn. Neurosci. 20, 342–355. 10.1162/jocn.2008.20024 18275339

[B42] Mutlu M. C. Erdogan S. B. Öztürk O. C. Canbeyli R. Saybaşιlι H. (2020). Functional near-infrared spectroscopy indicates that asymmetric right hemispheric activation in mental rotation of a jigsaw puzzle decreases with task difficulty. Front. Human Neurosci. 14, 252. 10.3389/fnhum.2020.00252 32694987 PMC7339288

[B43] Oruc I. Balas B. Landy M. S. (2019). Face perception: a brief journey through recent discoveries and current directions. Vision Res. 157, 1–9. 10.1016/j.visres.2019.06.005 31201832 PMC7371014

[B44] Özdem C. Wiese E. Wykowska A. Müller H. Brass M. OverwalleVan F. (2016). Believing androids – fMRI activation in the right temporo-parietal junction is modulated by ascribing intentions to non-human agents. Soc. Neurosci. 12, 582–593. 10.1080/17470919.2016.1207702 27391213

[B45] Parsons C. E. Stark E. A. Young K. S. Stein A. Kringelbach M. L. (2013). Understanding the human parental brain: a critical role of the orbitofrontal cortex. Soc. Neurosci. 8, 525–543. 10.1080/17470919.2013.842610 24171901

[B46] Peirce J. W. (2009). Generating stimuli for neuroscience using PsychoPy. Front. Neuroinform. 2:10. 10.3389/neuro.11.010.2008 19198666 PMC2636899

[B47] Rorden C. Brett M. (2000). Stereotaxic display of brain lesions. Behav. Neurol. 12, 191–200. 10.1155/2000/421719 11568431

[B48] Said C. P. Sebe N. Todorov A. (2009). Structural resemblance to emotional expressions predicts evaluation of emotionally neutral faces. Emotion 9, 260–264. 10.1037/a0014681 19348537

[B49] Scherer K. R. Moors A. (2019). The emotion process: event appraisal and component differentiation. Ann. Rev. Psychol. 70, 719–745. 10.1146/annurev-psych-122216-011854 30110576

[B50] Scholkmann F. Kleiser S. Metz A. J. Zimmermann R. Mata Pavia J. Wolf U.. (2014). A review on continuous wave functional near-infrared spectroscopy and imaging instrumentation and methodology. NeuroImage 85, 6–27. 10.1016/j.neuroimage.2013.05.004 23684868

[B51] Stearns S. D. Hush D. R. (2016). Digital Signal Processing with Examples in MATLAB^Ⓡ^. 10.1201/9781439837832

[B52] Strait M. Canning C. Scheutz M. (2014). Investigating the effects of robot communication strategies in advice-giving situations based on robot appearance, interaction modality and distance, in ACM/IEEE International Conference on Human-Robot Interaction (Bielefeld), 479–486.

[B53] Tinwell A. Grimshaw M. Nabi D. A. (2015). The effect of onset asynchrony in audio-visual speech and the uncanny valley in virtual characters. Int. J. Mech. Robot. Syst. 2, 97. 10.1504/IJMRS.2015.068991

[B54] Tuscan L. A. Herbert J. D. Forman E. M. Juarascio A. S. Izzetoglu M. Schultheis M. (2013). Exploring frontal asymmetry using functional near-infrared spectroscopy: a preliminary study of the effects of social anxiety during interaction and performance tasks. Brain Imaging Behav. 7, 140–153. 10.1007/s11682-012-9206-z 23132684

[B55] Uno T. Kawai K. Sakai K. Wakebe T. Ibaraki T. Kunii N.. (2015). Dissociated roles of the inferior frontal gyrus and superior temporal sulcus in audiovisual processing: top-down and bottom-up mismatch detection. PLoS ONE 10, e0122580. 10.1371/journal.pone.0122580 25822912 PMC4379108

[B56] Vos T. Flaxman A. D. Naghavi M. Lozano R. Michaud C. Ezzati M.. (2012). Years lived with disability (YLDs) for 1160 sequelae of 289 diseases and injuries 1990-2010: a systematic analysis for the global burden of disease study 2010. Lancet 380, 2163. 10.1016/S0140-6736(12)61729-2 23245607 PMC6350784

[B57] Wang Y. Quadflieg S. (2014). In our own image? emotional and neural processing differences when observing human-human vs human-robot interactions. Soc. Cogn. Affect. Neurosci. 11, 1515–1524. 10.1093/scan/nsv043 25911418 PMC4631149

[B58] Watanabe A. Kato N. Kato T. (2002). Effects of creatine on mental fatigue and cerebral hemoglobin oxygenation. Neurosci. Res. 42, 279–285. 10.1016/S0168-0102(02)00007-X 11985880

[B59] Wittfoth M. Schardt D. M. Fahle M. Herrmann M. (2009). How the brain resolves high conflict situations: double conflict involvement of dorsolateral prefrontal cortex. NeuroImage 44, 1201–1209. 10.1016/j.neuroimage.2008.09.026 18951983

[B60] Wolf R. C. Philippi C. L. Motzkin J. C. Baskaya M. K. Koenigs M. (2014). Ventromedial prefrontal cortex mediates visual attention during facial emotion recognition. Brain. 137, 177296-1780. 10.1093/BRAIN/AWU063 24691392 PMC4032099

[B61] Ye J. C. Tak S. Jang K. E. Jung J. Jang J. (2009). NIRS-SPM: Statistical parametric mapping for near-infrared spectroscopy. NeuroImage 44, 428–447. 10.1016/j.neuroimage.2008.08.036 18848897

[B62] Yorgancigil E. Urgen B. A. Yildirim F. (2021). Uncanny valley effect is amplified with multimodal stimuli and varies across ages. PsyArXiv [Preprints] 10.31234/OSF.IO/DTVJP

[B63] Yu J. Ang K. K. Ho S. H. Sia A. Ho R. (2017). Prefrontal cortical activation while viewing urban and garden scenes: a pilot fNIRS study, in Proceedings of the Annual International Conference of the IEEE Engineering in Medicine and Biology Society, EMBS (Jeju), 2546–2549. 10.1109/EMBC.2017.8037376 29060418

[B64] Yücel M. A. Selb J. Aasted C. M. Lin P.-Y. Borsook D. Becerra L.. (2016). Mayer waves reduce the accuracy of estimated hemodynamic response functions in functional near-infrared spectroscopy. Biomed. Opt. Express 7:3078. 10.1364/BOE.7.003078 27570699 PMC4986815

[B65] Zhao T. Chen J. Wang L. Yan N. (2020). Emotional appraisal processing of computer-generated facial expressions: an functional near-infrared spectroscopy study. NeuroReport 31, 437–441. 10.1097/WNR.0000000000001420 32168120

