# Supplementary figures and images for: An Exploratory Analysis of the Neural Correlates of Human-Robot Interactions With Functional Near Infrared Spectroscopy
Source: Front Hum Neurosci. 2022 Jul 18;16:883905. doi: 10.3389/fnhum.2022.883905 (PMC9339604; doi:10.3389/fnhum.2022.883905)

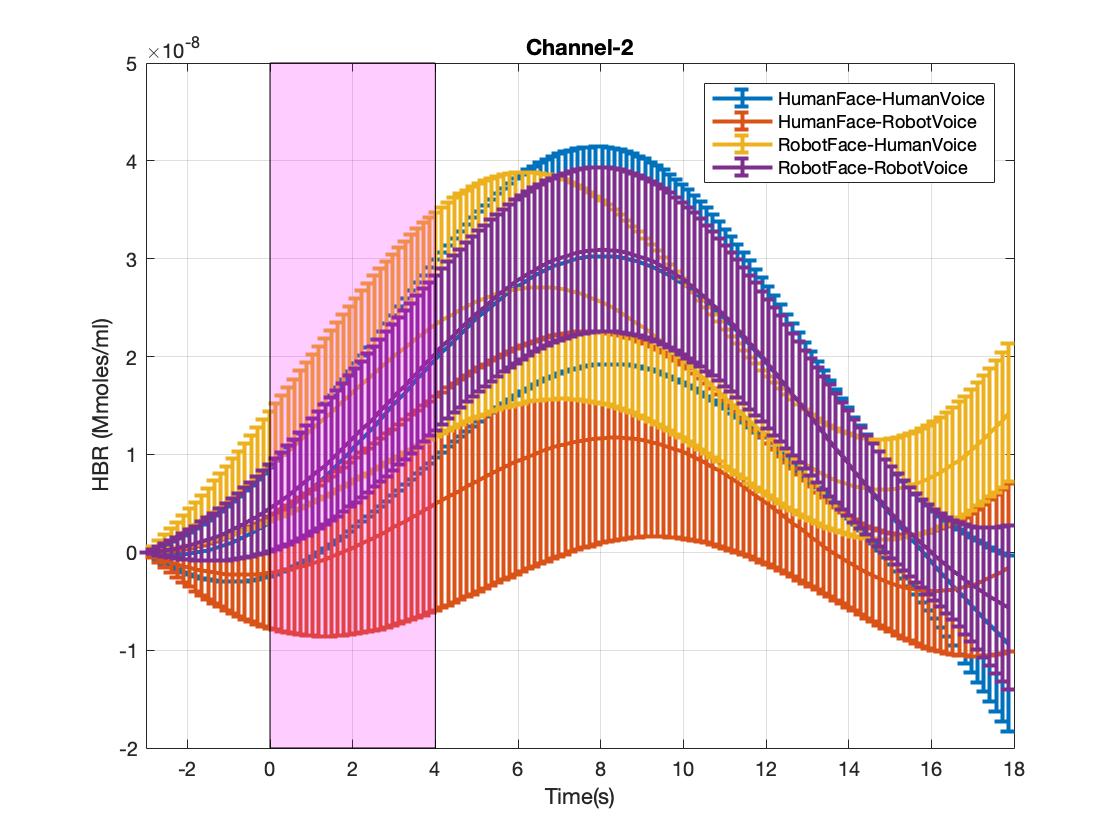

Supplement: Supplementary file 5 [file Data_Sheet_1.ZIP › HBR_Ch2.jpg]

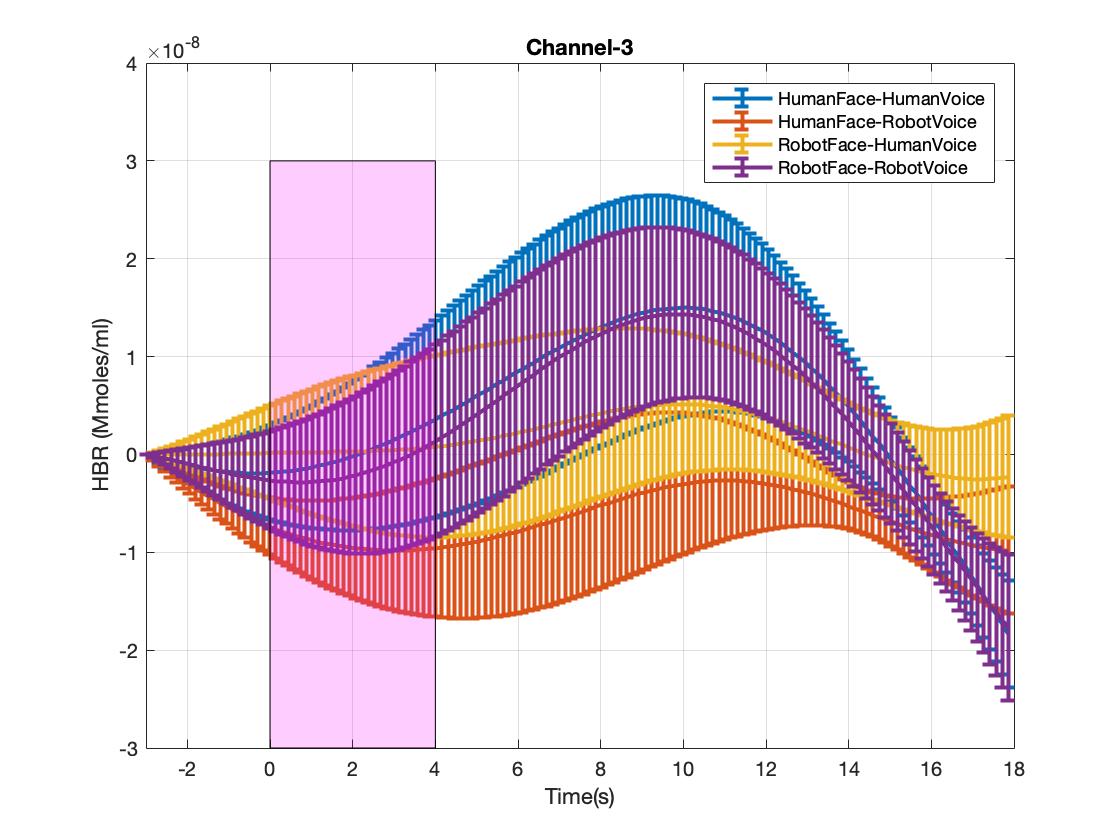

Supplement: Supplementary file 5 [file Data_Sheet_1.ZIP › HBR_CH3.jpg]

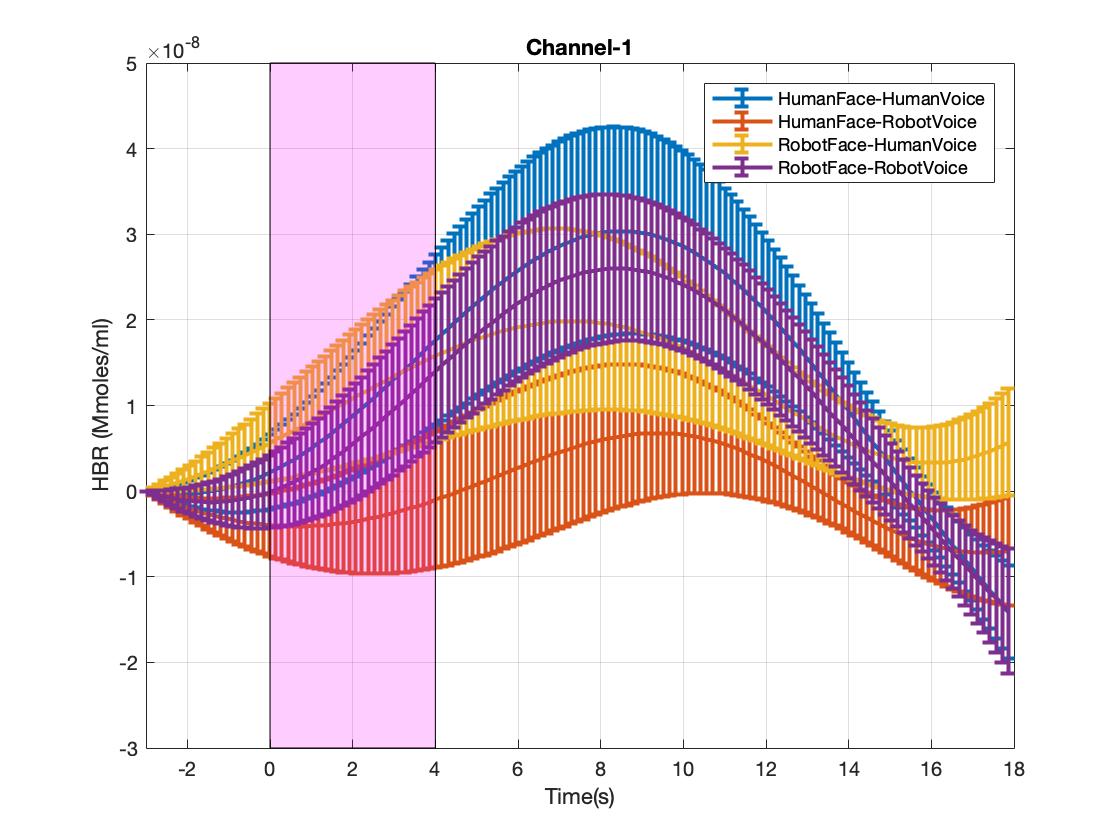

Supplement: Supplementary file 5 [file Data_Sheet_1.ZIP › HBR_Ch7.jpg]

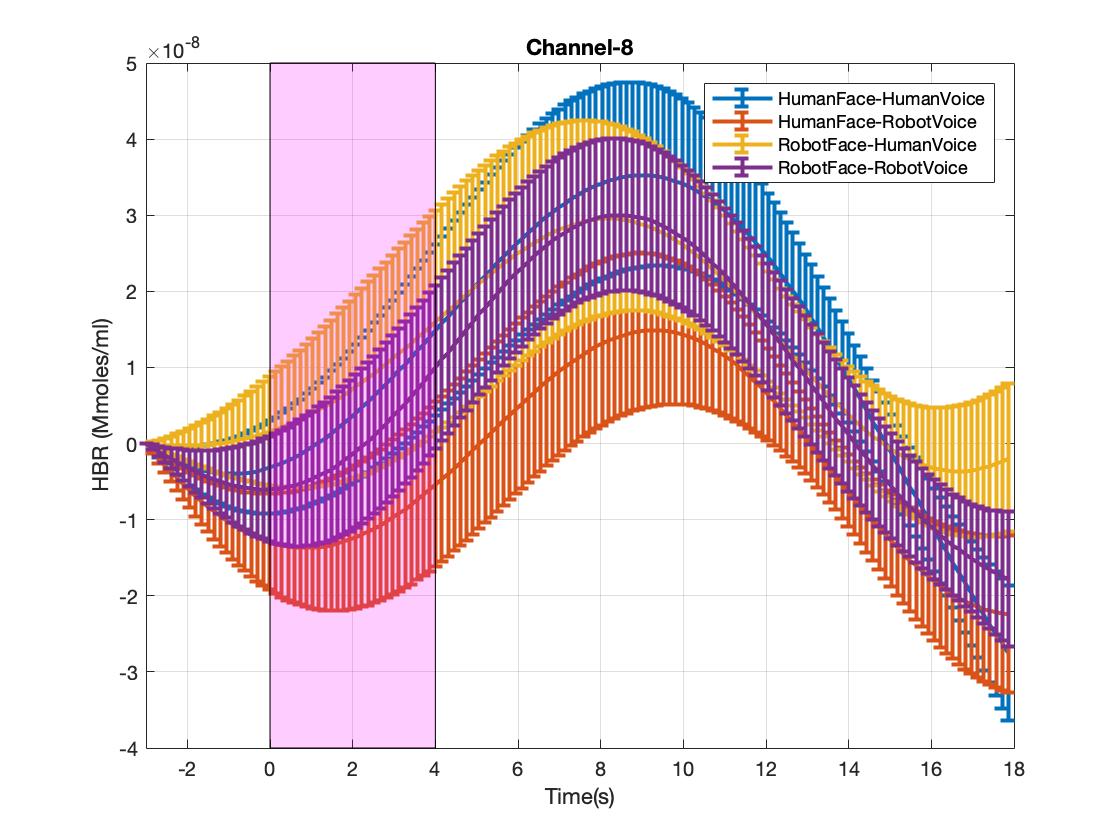

Supplement: Supplementary file 5 [file Data_Sheet_1.ZIP › HBR_Ch8.jpg]

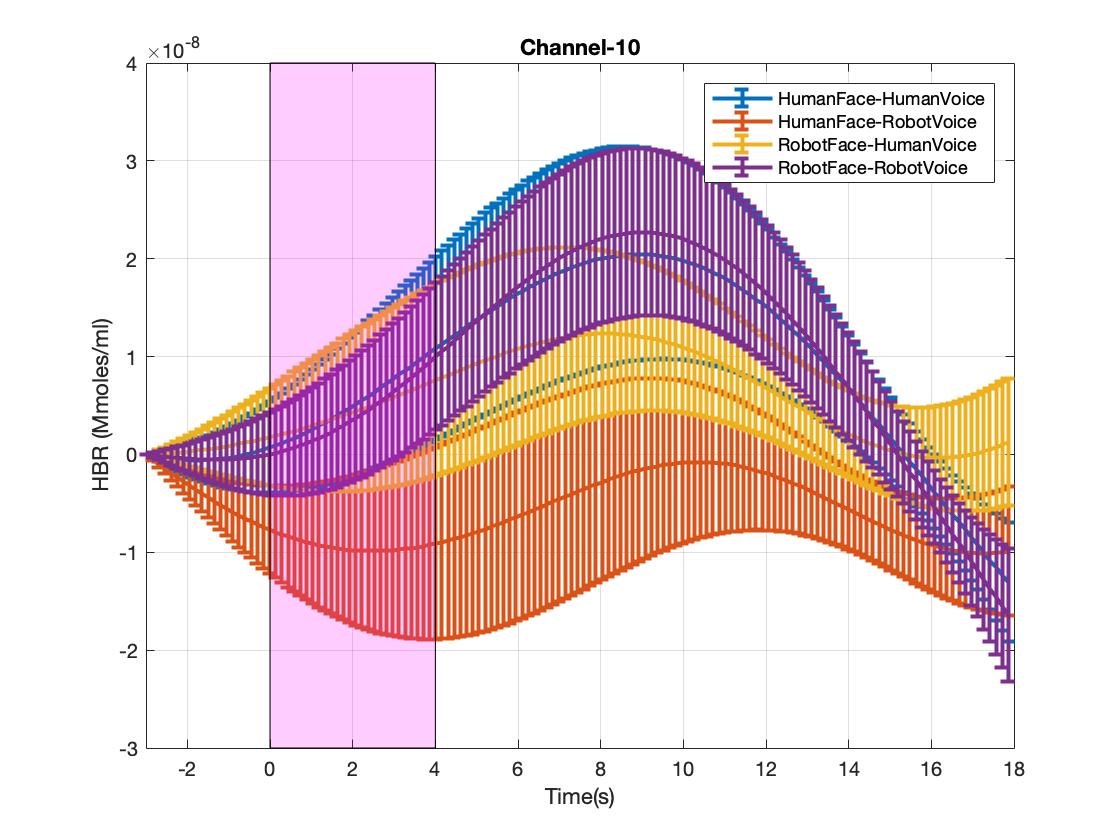

Supplement: Supplementary file 5 [file Data_Sheet_1.ZIP › HBR_Ch10.jpg]

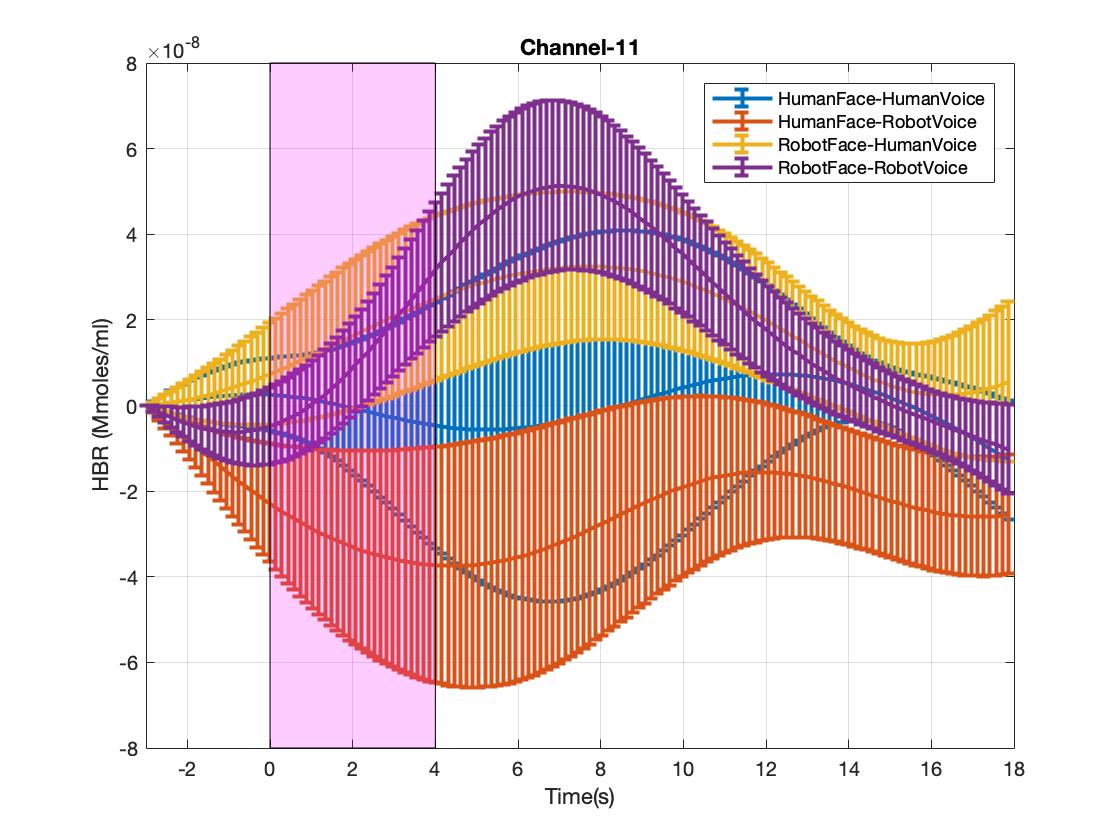

Supplement: Supplementary file 5 [file Data_Sheet_1.ZIP › HBR_Ch11.jpg]

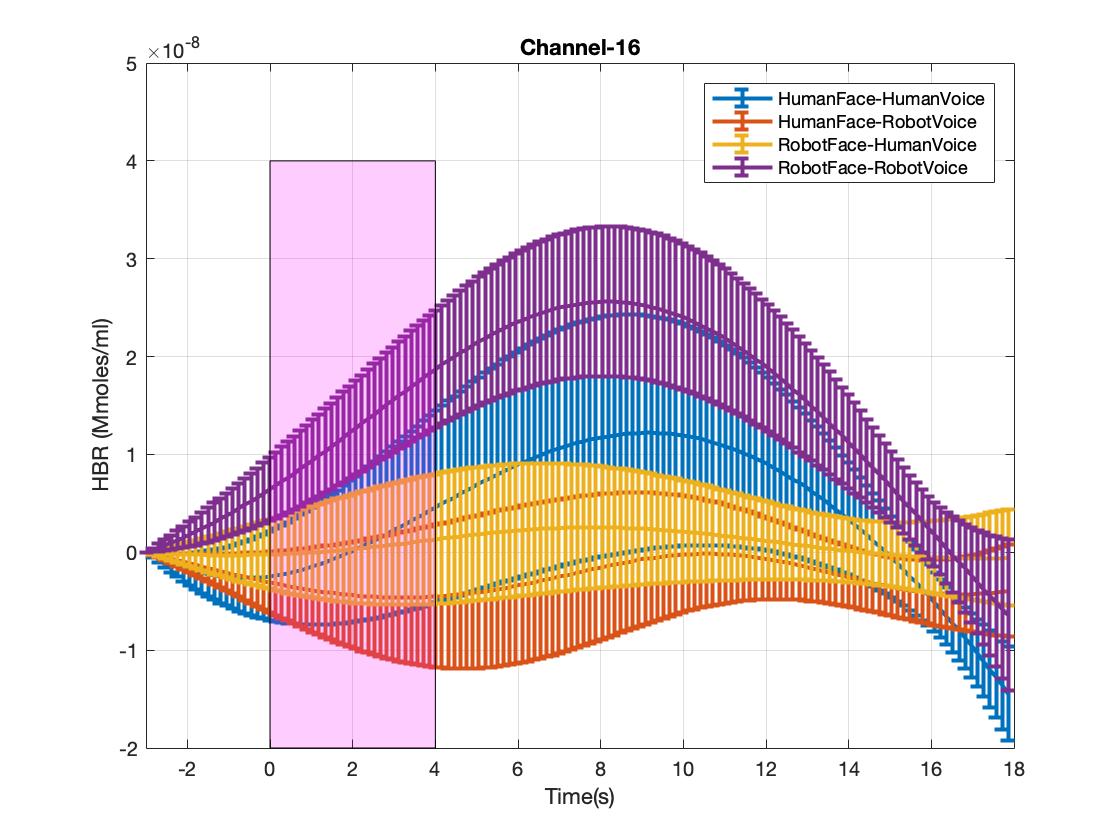

Supplement: Supplementary file 5 [file Data_Sheet_1.ZIP › HBR_Ch16.jpg]

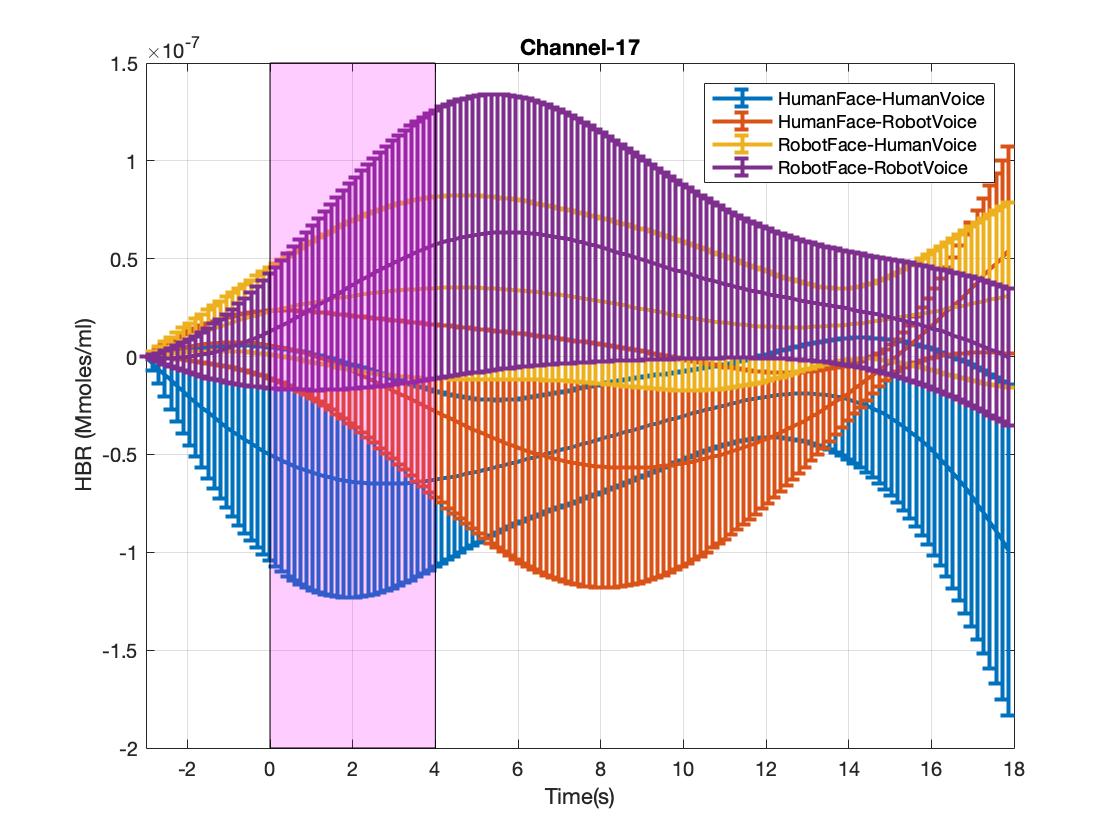

Supplement: Supplementary file 5 [file Data_Sheet_1.ZIP › HBR_Ch17.jpg]

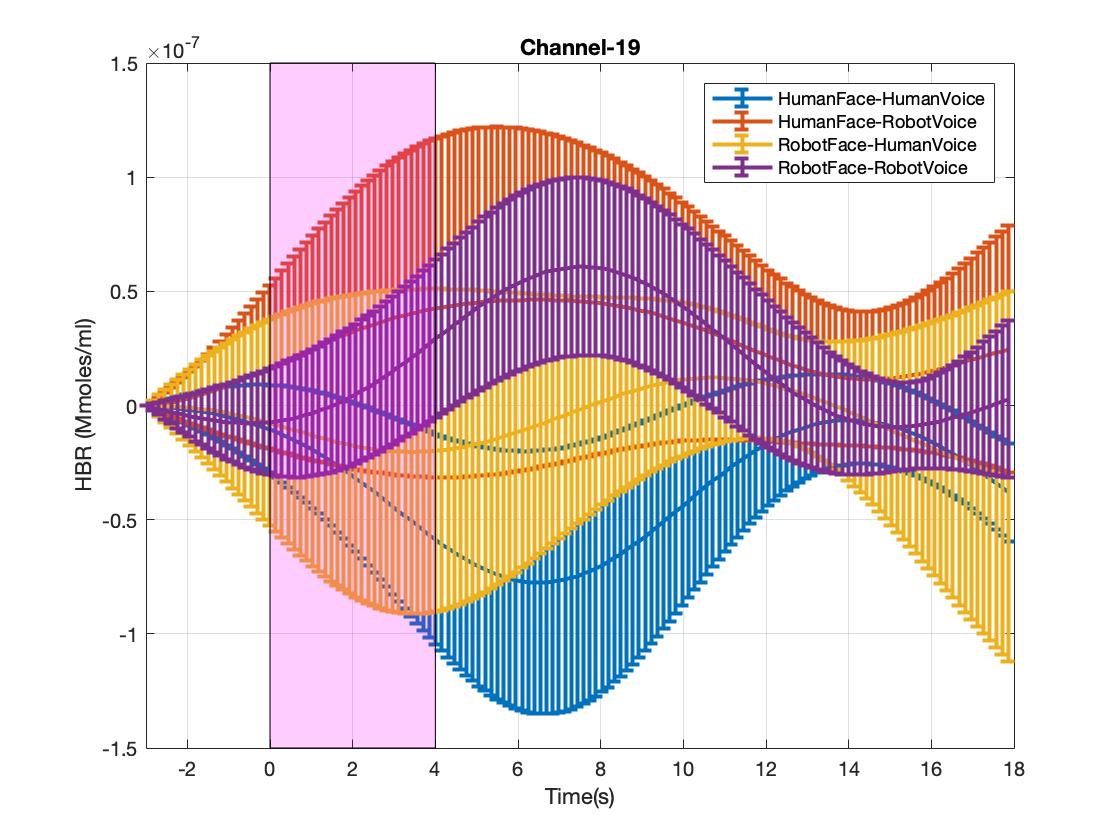

Supplement: Supplementary file 5 [file Data_Sheet_1.ZIP › HBR_Ch19.jpg]
